# Children Learning About Secondhand Smoke (CLASS II): A Pilot Cluster Randomized Controlled Trial

**DOI:** 10.1093/ntr/nty090

**Published:** 2018-05-16

**Authors:** Kamran Siddiqi, Rumana Huque, Mona Kanaan, Farid Ahmed, Tarana Ferdous, Sarwat Shah, Cath Jackson, Steve Parrott, Jasjit S Ahluwalia, Aziz Sheikh

**Affiliations:** 1 Department of Health Sciences, University of York Seebohm Rowntree Building, Heslighton, York, UK; 2 Department of Economics, University of Dhaka Social Science Building, Dhaka, Bangladesh; 3 ARK Foundation, Dhaka, Bangladesh; 4 Department of Health Sciences, University of York ARRC Building, University of York, Heslington, York, UK; 5 Brown University School of Public Health, Providence, RI; 6 Usher Institute of Population Health Sciences and Informatics, The University of Edinburgh Teviot Place, Edinburgh, UK

## Abstract

**Introduction:**

Children exposed to secondhand smoke (SHS) are at increased risk of respiratory illnesses. We piloted a Smoke Free Intervention (SFI) and trial methods before investigating its effectiveness and cost-effectiveness in primary school children.

**Methods:**

In a pilot cluster randomized controlled trial in Bangladesh, primary schools were allocated to usual education (control) or SFI, using minimization. Year-5 children were recruited. Masking treatment allocation was not possible. Delivered by schoolteachers, SFI consisted of two 45-min and four 15-min educational sessions. Our primary outcome was SHS exposure at two months post randomization, verified by children’s salivary cotinine. The trial is registered at ISRCTN.com; ISRCTN68690577.

**Results:**

Between April 1, 2015 and June 30, 2015, we recruited 12 schools. Of the 484 children present in Year-5, 481 consented. Six schools were allocated to both SFI (*n* = 245) and to usual education only (*n* = 236). Of them, 450 children (SFI = 229; control = 221) who had cotinine levels indicative of SHS exposure were followed-up. All schools were retained, 91% children (208/229) in SFI and 88% (194/221) in the control arm completed primary outcome assessment. Their mean cotinine at the cluster level was 0.53 ng/ml (*SD* 0.36) in SFI and 1.84 ng/ml (*SD* 1.49) in the control arm—a mean difference of −1.31 ng/ml (95% CI = −2.86 to 0.24).

**Conclusion:**

It was feasible to recruit, randomize, and retain primary schools and children in our trial. Our study, though not powered to detect differences in mean cotinine between the two arms, provides estimates to inform the likely effect size for future trials.

**Implications:**

In countries with high smoking prevalence, children remain at risk of many conditions due to secondhand smoke exposure. There is little empirical evidence on the effectiveness and cost-effectiveness of interventions that can reduce their exposure to secondhand smoke at homes. CLASS II trial found that a school-based intervention (SFI) has the potential to reduce children’s exposure to SHS—an approach that has been rarely used, but has considerable merit in school-based contexts. CLASS II trial provides key information to conduct a future definitive trial in this area of public health, which despite its importance has so far received little attention.

## Introduction

The health consequences of children’s exposure to secondhand smoke (SHS) are serious and well established.^1^ SHS exposure impairs their lung development and causes immune dysregulation.^2^ Children are therefore at an increased risk of chest infections, tuberculosis (TB),^3^ and asthma.^4^ Moreover, SHS exposure in children and adolescents leads to poor cognitive functions and academic achievements.^5^ Children exposed to smoking behaviors by their family members have an increased chance of taking up smoking.^6^ Unfortunately, 40% of children could be exposed to SHS worldwide amounting to a major public health threat.^7^

Signatory to the Framework Convention of Tobacco Control (FCTC), most countries have now banned smoking in indoor public spaces and workplaces.^8^ Where enforced strictly, these bans have resulted in a significant reduction in SHS exposure and its associated morbidity and mortality.^9^ However, children are mostly exposed to SHS in their homes and cars;^10^ additional measures are therefore required to provide comprehensive protection from SHS exposure.

Bangladesh, among the first few signatories to FCTC, introduced smoke-free legislation in 2005–2006 and strengthened it further in 2012 through a comprehensive smoking ban in most indoor public places, workplaces, and public transport.^11^ Despite this, the 2009 Global Adult Tobacco Survey (GATS) suggested that 57% of children (27.6 million) could be exposed to SHS in Bangladesh.^12^

There is little evidence on the effectiveness of interventions to protect children from SHS exposure. Two recent reviews remain inconclusive. A Cochrane review included 57 trials, many assessing the effect of parental education and counseling programs;^13^ a further systematic review and meta-analysis, included 16 trials of interventions delivered by healthcare professionals who provide routine child healthcare, neither found a significant reduction in children’s SHS exposure.^14^ Another meta-analysis, which reported on the effect of interventions aimed at reducing SHS exposure in homes, found some improvements but recommended further research.^15^

In Bangladesh, we developed a school-based Smoke Free Intervention (SFI) to encourage children to negotiate smoking restrictions in their households. In a feasibility trial (CLASS I), we found that SFI was successful in implementing self-reported voluntary smoking restrictions and in reducing social visibility of smoking behavior (*OR* = 3.9, 95% CI = 2.0 to 7.5) at home.^16^ However, we did not demonstrate if this change translated in a reduction in SHS exposure in children (eg, reduction in cotinine levels) or in indoor air pollution (eg, reduction in PM_2.5_).

Our ultimate aim is to assess the effectiveness and cost-effectiveness of the SFI in children exposed to SHS. Prior to conducting a definitive trial to answer the above question, we sought preliminary evidence of effectiveness in this population and tested methods for recruitment, randomization, and outcomes measurement. We also explored acceptability and feasibility of delivering the SFI with teachers and head teachers. This is reported elsewhere (manuscript in preparation).

## Methods

### Study Design

CLASS II was a large, two-arm, pilot, cluster randomized controlled trial (RCT) with an embedded preliminary economic analysis. It was conducted in 12 schools in Dhaka Division, Bangladesh. The study received ethics approvals from Bangladesh Medical Research Council’s and the University of York’s ethics committees. A detailed trial protocol has previously been published.^17^

### Participants

We recruited children from primary schools that followed national curricula, had Year-5 classes with >40 and <120 children/class, an associated secondary school, and a non-smoking policy on their premises. All schools situated within Mirpur and Savar (Dhaka) were contacted and those that responded positively within seven days were assessed for eligibility. All Year-5 children (expected age range = 10–12 years) who were self-reported non-smokers, were eligible to participate. We excluded children with serious mental and physical conditions, disabilities including learning difficulties, and those showing severe behavioral problems. The schools provided a list of all eligible children who were then recruited after obtaining their written, informed, assent, and parental consent on an opt-out basis.

### Randomization

All participating schools were randomly allocated to two arms following a computer-generated minimization sequence. Although schools in both arms received routine education as prescribed by the National Curriculum and Textbook, those in the intervention arm received SFI in addition. At the time of the CLASS II trial, this curriculum and the textbooks contained no information on secondhand smoke and its associated harms. Minimization—a method of adaptive stratified sampling—was used to restrict randomization on school’s public/private status and boys to girls’ ratio. The schools’ identification was concealed from the trial statistician, who generated the allocation sequence and assigned schools to the trial arms. Because of the nature of the intervention, it was not possible to mask the children, schoolteachers or researchers from the intervention allocation.

### Procedures

Soon after obtaining consent, we carried out children’s baseline assessment. This contained a classroom-administered questionnaire that included socio-demographic information and questions on smoking behavior, quality of life, and health service use and was completed by participating children. In addition, schoolteachers completed an Academic Performance Questionnaire (APQ)^18^ and a school absenteeism form. We also assessed children’s lung function tests and collected saliva (morning samples) for cotinine test. Children were also asked to complete a daily respiratory symptoms diary. After baseline data collection, schools were allocated to the two trial arms. Post allocation, children with salivary cotinine levels indicative of SHS exposure were followed-up at 2, 6, and 12 months. The follow-ups included all assessments except salivary cotinine levels, which happened at 2-month follow-up only.

### Intervention

SFI was a theory-based behavior change intervention^19^ developed by a multidisciplinary group in Bangladesh.^16^ SFI was delivered by Year-5 schoolteachers who were provided the relevant resource materials and training. The intervention was delivered to all children as a group in a classroom setting irrespective of their baseline cotinine levels. It consisted of two 45-min sessions delivered over two consecutive days. Each session included classroom presentations, quiz, interactive games, storytelling, and role-play—utilizing vicarious learning techniques.^16^ The presentation, quiz, and games aimed to make children aware of the harms of SHS and motivate them to achieve a smoke-free home. The storytelling and role-play activities focused on building children’s confidence in raising their concerns about SHS with their parents and enhance their negotiation skills. Although storytelling illustrated numerous challenges of discussing adults’ smoking behavior within families, role-play allowed children to learn and practice relevant negotiating strategies. These were followed by four refresher sessions (15 min each) over the subsequent 4 weeks. These sessions reinforced learning by revising the salient points of the initial sessions and by encouraging children to share their experience of initiating relevant conversations within their families. Teachers also helped children to plan their next action. Children were also provided with take-home promise forms for families that provided graphic representations of the hazards of SHS, pictorial guidance to help them make their homes smoke free, and a tear-off slip to commit to imposing smoking restrictions at home. These restrictions extended to visitors and cars too. Teachers were also trained to pick up any signs of distress among children as an untoward consequence of SFI.

### Controls

Schools in the control arm did not receive any intervention but only study information.

### Outcomes

The primary outcome was a change in children’s salivary cotinine—a sensitive biochemical marker strongly associated with recent SHS exposure. Collected by keeping a sterile swab in the mouth for approximately 5 min and then transferring to a sterile plastic container, the saliva samples were analyzed using gas-liquid chromatography technique at ABS laboratories in the United Kingdom.

Our secondary outcomes included the frequency and severity of respiratory symptoms, lung function tests, self-reported smoking restrictions, health service use, quality of life, academic performance, and school absenteeism.

Furthermore, children kept a diary for 13 respiratory symptoms and recorded their severity on a four-point scale on a daily basis.^20^ For upper respiratory tract symptoms, children reported on having a runny nose or sneezing, blocked or stuffy nose, sore throat or hoarse voice, headaches or face aches, aches or pains elsewhere, and feeling chill, fever, or shivers. For lower respiratory symptoms, cough on waking, wheeze on waking, cough during the day, wheeze during the day, shortness of breath during the day, night cough, and wheeze or shortness of breath during the night, were included. Presence of at least four of these symptoms on any one day was considered a clinical episode. We estimated the proportion of children with at least one clinical episode and mean clinical episodes per child. Children’s lung functions including forced vital capacity (FVC), forced expiratory volume in the first second (FEV1), and peak expiratory flow (PEF) were measured using a handheld Micro1 spirometer as per Thoracic Society guidelines.^21^ In addition to absolute PEF, we estimated relative PEF as a proportion of the predicted PEF, based on age and sex.

Children reported on smoking restrictions and its social visibility at home. The questionnaire asked: (1) “Are people who live with you allowed to smoke?” (Anywhere inside your home/in some rooms in your home/ only in one room in your home/ only outside); (2) “Are people who visit your home allowed to smoke?” (Anywhere inside your home/in some rooms in your home/ only in one room in your home/ only outside); (3) “Are people who live with you allowed to smoke in front of children?” (Y/N); and (4) “Are people who visit your home allowed to smoke in front of children?” (Y/N). Variables on smoking restrictions for residents and visitors were later combined to create a composite variable indicating “complete restriction” if the responses were “only outside” for both variables, “no restriction” if the answer was “anywhere inside your home” for either of the two variables and “partial restriction” for all other combinations.

Children’s academic performance was assessed using the Academic Performance Questionnaire (APQ).^18^ The teachers reported on children’s reading, maths and writing performance as: (1) well above average; (2) at or somewhat above average; (3) somewhat below average; and (4) well below average. Schools also provided reports on children’s school absenteeism (number of days missed in the last month).

We also measured recruitment and attrition rates for clusters and participants including their reasons for ineligibility and non participation, the intra-cluster correlation coefficient (ICC) for the primary outcome, the costs associated with delivering SFI, and the time and resources required in measuring outcomes and the extent and type of missing data. The baseline questionnaire also collected data on other potential confounders including: age, gender, medical history, household amenities, family structure, co-habiting smokers—including parents, pet ownership, overcrowding—number of rooms and residents, built environment, neighborhood (number of shops selling tobacco within 5 min of walking distance from home), presence of mould/moisture, and the type of fuel used for cooking in homes.

### Statistical Analysis

We aimed to recruit at least 12 schools (clusters) and 360 children (participants), 30 per school, for this pilot RCT. Among these, we expected at least a third (120 children) to have a baseline salivary cotinine result indicative of recent SHS exposure. Based on our feasibility study,^16^ we predicted retaining all 12 schools and at least 80% of all participants in the trial. We anticipated that a pilot trial that retains approximately 100 children was likely to provide robust estimates of the effect size, recruitment and retention rates, and ICC ahead of a definitive trial.

We conducted a preliminary analysis summarizing: participant (individual and cluster) characteristics, recruitment attrition rates, effect size, and ICC. Although determining differences in the outcomes between the two arms was not the purpose of this study, we summarized outcomes at both cluster and individual levels using an intention-to-treat principle (ITT) and estimated 95% confidence intervals (CIs) for any differences. We explored the likely difference in the cotinine levels at the first follow-up at the cluster level using two-sample *t*-test adjusted for unequal variance. Furthermore, we explored the likely effect size using individual level data adjusted for clustering and taking into account minimization variables. To this effect, we have used linear regression models with random intercepts to account for the clustering using maximum likelihood estimation. We also report on the effect size when controlling for baseline cotinine levels and other demographic baseline variables. We also summarized all other secondary outcomes descriptively. All analyses were conducted using STATA v.14.^22^

### Preliminary Economic Analysis

We also assessed the feasibility of undertaking a full cost-effectiveness analysis in future. We estimated the cost of delivering the SFI including the time and resources needed to deliver the intervention. Health service utilization was assessed by asking pre-tested questions^23^ on contacts with doctors/nurses, hospital admissions, pharmacy visits, and antibiotic prescriptions. Quality of life was assessed using a short quality of life questionnaire for children EQ-5DY.^24^ We also audited data for completeness.

The trial is registered at ISRCTN.com and the number ISRCTN68690577.

## Results

**Figure 1.**
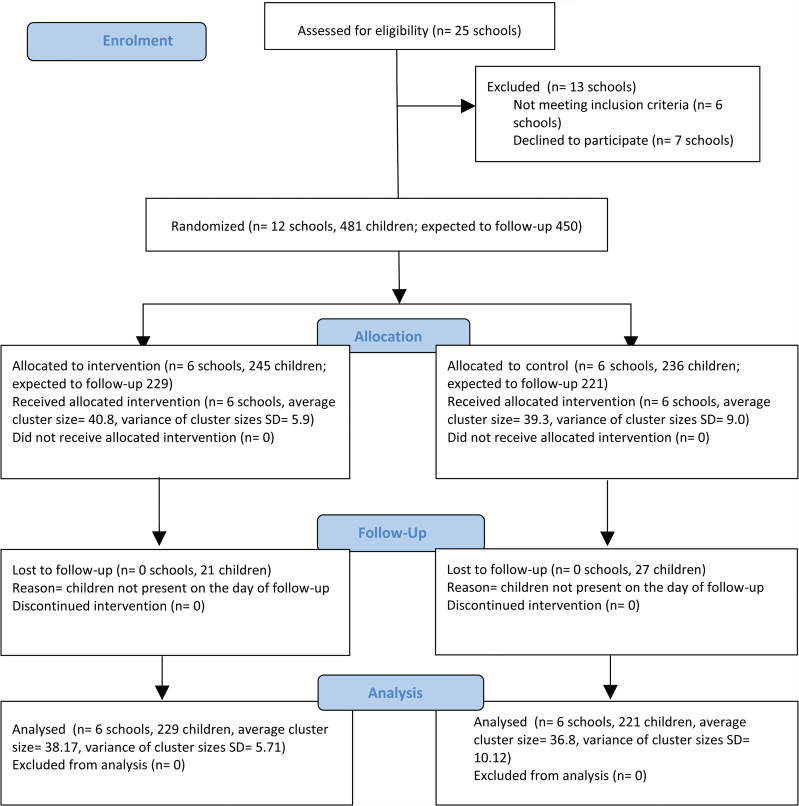
CLASS II Trial Flow Diagram (adapted from CONSORT 2010).

Between April 1, 2015 and June 30, 2015, we approached 25 schools and recruited 12 (Figure 1); seven declined to participate due to workload issues and six were ineligible: three schools did not follow government-approved curriculum, one had a small class size, and two were not linked to secondary schools. Of 576 children studying in Year-5 in the 12 schools, 484 were present on the recruitment day; 481 consented whereas three declined without giving any specific reasons.

Six schools (245 children) were allocated to the intervention and six (236 children) to the control arm. All 12 schools were retained throughout the trial. Based on the baseline cotinine (indicative of SHS exposure), we expected to follow-up 450 children (229 in the intervention and 221 in the control arm). However, 91% children (208/229) in the intervention and 88% (194/221) in the control arm completed their first follow-up at which the primary outcome was assessed. Similarly, 95% (217/229) and 94% children (215/229) in the intervention and 92% (203/221) and 91% children (201/221) in the control arm completed their 6-month and 12-month follow-ups, respectively.

Both arms were balanced on key sociodemographic and environmental characteristics (Table 1). The majority of children lived in homes with an outdoor space (average % per cluster 68) and used clean fuels for cooking (average % per cluster 94), but had no visible mould damage (average % per cluster 59) or kept any cattle (average % per cluster 78). However, there were baseline differences between the two arms on family smoking patterns, that is, living with a smoker and smoking restrictions at home (Tables 1 and 3).

**Table 1.** Baseline Characteristics of Clusters and Children Retained in the Trial

	Individual level			Cluster level		
	Control	Intervention	Total	Control	Intervention	Total
	(*N* = 221)	(*N* = 229)	(*N* = 450)	(*N* = 6)	(*N* = 6)	(*N* = 12)
	*n* (%)*	*n* (%)*	*n* (%)*	average %* per cluster	average %* per cluster	average %* per cluster
Girls	115 (52.0)	117 (51.1)	232 (51.6)	51.2	51.2	51.2
Boys	106 (48.0)	112 (48.9)	218 (48.4)	48.8	48.8	48.8
Age (years): Mean (SD)	10.9 (1.1)	10.8 (1.3)	10.8 (1.2)	10.8 (0.3)	10.8 (0.4)	10.8 (0.3)
**Lives with a smoker**						
No	111 (50.2)	143 (62.5)	254 (56.4)	48.6	62.8	55.7
Yes	110 (49.8)	86 (37.5)	196 (43.6)	51.4	37.2	44.3
**Maternal/female carer education**						
No education	39 (17.7)	29 (12.7)	68 (15.1)	17.3	12.5	14.9
Primary	75 (34.0)	72 (31.4)	147 (32.7)	32.9	30.9	31.9
Secondary	76 (34.4)	90 (39.3)	166 (36.9)	34.1	38.9	36.5
Higher education	31 (14.0)	38 (16.6)	69 (15.3)	15.6	17.8	16.7
**Paternal/male carer education**						
No education	23 (10.4)	19 (8.3)	42 (9.3)	9.4	8.1	8.8
Primary	68 (30.8)	46 (20.1)	114 (25.3)	30	20.2	25.1
Secondary	69 (31.2)	95 (41.5)	164 (36.4)	31.8	40.9	36.4
Higher education	61 (27.6)	69 (30.1)	130 (28.9)	28.7	30.8	29.8
**Home with an outdoor space**						
No	57 (25.8)	90 (39.3)	147 (32.7)	25.4	38.6	32
Yes	164 (74.2)	139 (60.7)	303 (67.3)	74.6	61.4	68
**Type of fuel used for cooking**						
Electricity/clean fuels	200 (90.5)	221 (96.5)	421 (93.6)	91.5	96.4	93.9
Coal/Wood/Biomass	21 (9.5)	8 (3.5)	29 (6.4)	8.5	3.6	6.1
**Condition of the home (mould, damp**)						
No damage	143 (64.7)	125 (54.6)	268 (59.6)	65.5	53.4	59.4
Mould odour	1 (0.4)	1 (0.4)	2 (0.4)	3.7	4.5	4.1
Visible mould ± odor	11 (5.0)	3 (1.3)	14 (3.1)	5	1.4	3.2
Damp stains	26 (11.8)	51 (22.3)	77 (17.1)	11.9	22.9	17.4
Structural damage	40 (18.1)	49 (21.4)	89 (19.8)	17.3	21.9	19.6
**Cattle in homes**						
No	161 (72.9)	186 (81.2)	347 (77.1)	74.5	80.9	77.7
Yes	60 (27.1)	43 (18.8)	103 (22.9)	25.5	19.1	22.3
**Tobacco selling shops in vicinity:** Mean (SD)	4.9 (3.8)	5.1 (2.9)	5.0 (3.4)	5.2 (1.7)	5.0 (1.1)	5.1 (1.4)
**Cotinine**						
Mean (SD)	0.686 (1.05)	0.533 (0.629)	0.608 (0.866)	0.705 (0.161)	0.522 (0.275)	0.613 (0.235)

*Unless otherwise stated.

Out of 450 children, 402 provided a salivary sample for cotinine, two months postallocation. At 2-month follow-up, mean salivary cotinine at the cluster level was 0.53 ng/ml (*SD* 0.36) in the intervention arm compared to 1.84 ng/ml (*SD* 1.49) in the control providing a mean difference of −1.31 ng/ml (95% CI = −2.86 to 0.24) (Table 2). After adjusting for clustering, baseline cotinine, and other potential confounders, a similar mean difference of −1.54 ng/ml (95% CI = −3.47 to 0.38) was estimated.

**Table 2.** Estimates of the Primary Outcome (Saliva Cotinine) at 2 Months in Those Whose Saliva Cotinine Were Indicative of SHS Exposure at the Baseline

	Intervention			Control			Mean difference	
	*N*	Mean	*SD*	*N*	Mean	*SD*	(95% CI)	ICC
Salivary cotinine (at the individual level)	208	0.53	1.03	194	2.02	12.6		0 (0, 0.025)
Salivary cotinine (at the cluster level)	6	0.53	0.36	6	1.84	1.49	−1.31 (−2.86 to 0.24)**ρ**	
Salivary cotinine*							−1.32 (−3.28 to 0.64)	
Salivary cotinine**							−0.82 (−2.68 to 1.03)	
Salivary cotinine***							−1.33 (−3.25 to 0.59)	
Salivary cotinine****							−**1.54** (−**3.47 to 0.38**)	

**ρ** using two-sample *t*-test adjusted for unequal variance at the cluster level.

*Adjusted for clustering using individual level data and taking into account minimization variables.

** Adjusted for clustering and baseline cotinine using individual level data and taking into account minimization variables.

*** Adjusted for clustering, baseline cotinine, outside space, parental education levels, and tobacco shops in the neighborhood using individual level data and taking into account minimization variables.

**** Adjusted for clustering, baseline cotinine, smokers living in the house at baseline, outside space, parental education levels, and tobacco shops in the neighborhood using individual level data and taking into account minimization variables.

Table 3 and Supplementary Table 1 present a range of behavioral, clinical, and educational outcomes, respectively. No obvious differences between the intervention and the control arms were observed. Four-fifth of children in the intervention and three-fifth in the control arm reported complete smoking restrictions at home; little change was observed in both arms at the follow-ups. The average PEF per cluster remained a little below the predicted PEF (range = 83%–89%) in children in both trial arms at all timepoints. Almost all measures of educational attainment improved as the year progressed albeit in both arms. The proportion of children completing the respiratory symptom diary dropped from 70% and 72% in the first 2 months to 56% and 64% in the last 6 months in the intervention and the control arms, respectively (Supplementary Table 2). Most children (range = 82%–92%) recorded respiratory symptoms that reached clinical threshold at least once during all three time periods. Children, who reported symptoms with a score above the clinical threshold, did so for nearly half of the number of weeks in the first 6 months and for one-third of the number of weeks in the last 6 months. Children and teachers reported no adverse events despite specific enquiries at the follow-ups.

**Table 3.** Descriptive Statistics of the Behavioral and Clinical Outcomes at the Cluster Level at Baseline, 2-Month, 6-Month, and 12-Month Follow-ups

		Intervention				Control			
Outcomes*		Baseline	2 months	6 months	12 months	Baseline	2 months	6 months	12 months
Number of children		229	209	217	215	221	194	203	201
Number of clusters		6	6	6	6	6	6	6	6
Smoking restrictions at home	Complete	0.84 (0.084)	0.74 (0.129)	0.76 (0.141)	0.88 (0.029)	0.61 (0.158)	0.61 (0.122)	0.73 (0.089)	0.61 (0.171)
	Partial	0.03 (0.036)	0.09 (0.063)	0.10 (0.078)	0.04 (0.029)	0.06 (0.033)	0.10 (0.065)	0.11 (0.061)	0.15 (0.093)
	None	0.13 (0.077)	0.17 (0.096)	0.14 (0.102)	0.07 (0.045)	0.34 (0.150)	0.29 (0.143)	0.16 (0.065)	0.24 (0.166)
Lung function	Mean percentage of predicted PEF	88.3 (4.93)	89.1 (5.8)	85.8 (2.34)	83.7 (2.32)	85.8 (2.76)	88 (3.19)	83.2 (3.2)	85 (1.67)
Number of days absent (during the study period)	Mean	1.31 (0.257)	2.03 (1.2)	3.27 (0.34)	—	4.7 (6.9)	2.03 (1.2)	3.1 (0.377)	—

*Mean (SD) are reported at the cluster level.

### Preliminary Economic Analysis

Costs for the training were 56440 Bangladeshi taka (BDT) (equivalent to £536) in Mirpur and 42840 (£401) BDT at Savar giving a total of 99280 BDT (£943). Based on the 245 children in the intervention group the cost per child was 405.22 BDT (£3.85).

Based on two 45-min sessions and four 15-min refresher sessions, using a unit cost of 645.83 BDT per hour for teachers, the total intervention cost for the six schools was 9687 BDT—39.5 BDT (£0.38) per child. Summing training costs and intervention cost to derive the total cost per child was estimated at 444.72 BDT (£4.22).

EQ-5D-Y was administered at baseline and three follow-up timepoints (Supplementary Table 3a). There is no single utility weight that can be applied to EQ-5D-Y hence we report percentages reporting no problems in any of the five dimensions. In the overall sample at baseline, 53% reported no problems, compared with 59% at 2-month follow-up, 52% at 6-month follow-up, and 56% at 12-month follow-up. Complete EQ-5DY data (all items) and healthcare utilization data were returned for all individuals. The results showed very low rates of contact with healthcare services (Supplementary Table 3b and c). Only the GP visit and prescription categories showed attendance rates of above 5%. This suggests that collecting more detailed primary care and other parallel systems (pharmacies/traditional healers) utilization rates may be appropriate in a full RCT.

## Discussion

Given that CLASS II was a pilot trial, we cannot interpret its findings to make any definitive conclusions. However, the direction and magnitude of the effect size indicates that conducting a definitive trial to assess the effectiveness of SFI would be worthwhile. Our study provides key information to design and conduct such a trial in Bangladesh. We were able to recruit sufficient primary schools and retain all of them in the trial. Almost all children were eligible and able to participate; we were able to follow-up almost 90% children over a year. We were also able to assess primary and secondary outcomes for most of the children.

SFI relies on children’s motivation and ability to persuade their families to change their smoking behavior by highlighting its ill effects. Therefore, SFI makes two sequential assumptions: schoolteachers can encourage children to negotiate changes in their family’s smoking behavior, and this can motivate families to change. Often termed as “pester power,”^25^ the food and beverages industry have been using this to make families change their purchasing habits, but it has been rarely used in health promotion. The recent reviews on intervention to protect children from SHS exposure^13,26^ and to promote smoke-free homes^15,27^ did not include assessment of “pester power” to change family’s smoking behavior. The Cochrane reviews^13,28^ on the same topic included two school-based studies; one of which included schools that implemented smoke-free policies and asked children to persuade their families to do the same.^29^ Further exploration is needed to assess the potential of this approach in other health promotion interventions.

CLASS II trial had some limitations. Salivary cotinine only measures changes in children’s recent exposure to SHS. Moreover, we did not measure homes’ indoor air pollution levels—a future trial should include such measures. It is difficult to say if the difference observed in our study was a consequence of any smoking restrictions at home or due to other changes in smoking behaviors. Given that children were also exposed to SHS in places other than homes (Out of 95% children who were cotinine positive, only 44% lived with smokers), the change could be a reflection of children’s attempt to avoid places where people are visibly smoking. Although children were asked to report on smoking restrictions, self-reports in children are not validated and may not help in seeking explanations. Furthermore, we did not assess change in salivary cotinine at 6 and/or 12 months. We did not ask and exclude children on the basis of their smokeless tobacco use, which might have impacted our primary outcome of salivary cotinine. A future definitive trial should ask and exclude such children from the trial. Our intervention is complex and using several behavior change techniques; it would require a longitudinal evaluation to study processes/interactions—a consideration for future studies. Although children did not report any adverse consequences of negotiating smoking restrictions, it is possible that our intervention might have posed some difficulties for children. One potential criticism of SFI is that it makes children—the victims and not the cause of SHS—responsible for stimulating behavior change in adults. This potential burden of responsibility and its consequences require careful exploration in process evaluations.

CLASS II trial included a range of outcomes based on children’s lung health and their academic performance—a major strength. However, it did not assess adult smoking cessation. Furthermore, it also did not investigate the effect of SFI on children’s smoking uptake rates. Both of these outcomes are plausible and could be included in a future trial. Our assessment of the frequency and severity of respiratory symptoms using a daily diary saw a downward trend in children’s response rate at subsequent follow-ups. This particular assessment put children under a substantial research burden affecting data completeness and its potential accuracy. In future trials, we suggest assessing respiratory symptoms at intervals, for example, the first week of each month.

Although the tools used for assessing self-reported measures in our trial have been used previously in children, we acknowledge that the translation of these tools to Bangla might have influenced their validity. Further psychometric analysis is, therefore, warranted to assess their validity in Bangla. Likewise, we collected demographic information from children but we did not validate these from other sources. In a future trial, we suggest validating these responses from their parents/carers. A future trial should also consider stratified randomization using key behavioral variables (living with a smoker and smoking restrictions at home) to achieve a better balance across the trial arms than our pilot trial.

CLASS II is a pilot trial and therefore cannot make policy recommendations. However, its findings are highly relevant. It found that 95% of participating children were exposed to SHS. If true for other children in Bangladesh, this requires urgent and strong policy measures. The level of engagement shown by the schoolteachers and children was indicative of their willingness to take part in health promotion—relevant for delivering other public health measures through schools.

In summary, the CLASS II trial was successful in recruiting, retaining, and randomizing primary schools and collecting useful outcomes data from their Year-5 pupils in Bangladesh. We have shown that conducting a definitive trial in future to assess the clinical and cost-effectiveness of SFI is feasible and desirable.

## Funding

Medical Research Council (MR/M020533/1).

## Contributions

KS conceived the study, designed its protocol, interpreted the study findings, and wrote the first draft of the manuscript. RH coordinated the overall study implementation and commented on the draft manuscript. MK did the statistical analysis for the study and commented on the written manuscript. CJ contributed to the design of the protocol, was a co-investigator and commented on drafts of the manuscript. FA coordinated field data collection, supported data entry and data analyses, and commented on drafts of the manuscript. TF contributed in checking data completeness, provided support in statistical analysis, and commented on draft of the manuscript. SS contributed to the design of the protocol and commented on drafts of the manuscript. SP designed and conducted preliminary economic analysis and contributed to the manuscript. JSA provided written feedback on a number of drafts of the manuscript. AS helped secure funding, was a co-investigator and commented on several drafts of the manuscript.

## Declaration of Interests

We declare no competing interests.

## Supplementary Material

Supplementary Material

## References

[1] (US) OoSaH. The Health Consequences of Involuntary Exposure to Tobacco Smoke: A Report of the Surgeon General. Atlanta (GA): Centers for Disease Control and Prevention (US); 2006. 20669524

[2] Gibbs K, Collaco JM, McGrath-Morrow SA Impact of tobacco smoke and nicotine exposure on lung development. Chest. 2016;149(2):552–561. doi:10.1378/chest.15-1858. 26502117 10.1378/chest.15-1858 PMC4944770

[3] Dogar OF, Pillai N, Safdar N, Shah SK, Zahid R, Siddiqi K Second-hand smoke and the risk of tuberculosis: A systematic review and a meta-analysis. Epidemiol Infect. 2015;143(15):3158–3172. doi:10.1017/S0950268815001235. 26118887 10.1017/S0950268815001235 PMC9150979

[4] Dick S, Friend A, Dynes K, et al A systematic review of associations between environmental exposures and development of asthma in children aged up to 9 years. BMJ Open. 2014;4(11):e006554. doi:10.1136/bmjopen-2014-006554. 10.1136/bmjopen-2014-006554 PMC4244417 25421340

[5] Chen R, Clifford A, Lang L, Anstey KJ Is exposure to secondhand smoke associated with cognitive parameters of children and adolescents?–A systematic literature review. Ann Epidemiol. 2013;23(10):652–661. doi:10.1016/j.annepidem.2013.07.001. 23969303 10.1016/j.annepidem.2013.07.001

[6] Leonardi-Bee J, Jere ML, Britton J Exposure to parental and sibling smoking and the risk of smoking uptake in childhood and adolescence: A systematic review and meta-analysis. Thorax. 2011;66(10):847–855. doi:10.1136/thx.2010.153379. 21325144 10.1136/thx.2010.153379

[7] Oberg M, Jaakkola MS, Woodward A, Peruga A, Prüss-Ustün A Worldwide burden of disease from exposure to second-hand smoke: A retrospective analysis of data from 192 countries. Lancet. 2011;377(9760):139–146. doi.10.1016/S0140-6736(10)61388-8. 21112082 10.1016/S0140-6736(10)61388-8

[8] Frazer K, Callinan JE, McHugh J, et al Legislative smoking bans for reducing harms from secondhand smoke exposure, smoking prevalence and tobacco consumption. Cochrane Database Syst Rev. 2016;2:CD005992. doi:10.1002/14651858.CD005992.pub3. 26842828 10.1002/14651858.CD005992.pub3 PMC6486282

[9] Been JV, Nurmatov UB, Cox B, Nawrot TS, van Schayck CP, Sheikh A Effect of smoke-free legislation on perinatal and child health: A systematic review and meta-analysis. Lancet. 2014;383(9928):1549–1560. doi:10.1016/S0140-6736(14)60082-9. 24680633 10.1016/S0140-6736(14)60082-9

[10] Physicians RCo. Passive smoking and children: A report by the Tobacco Advisory Group of the Royal College of Physicians. London: Royal College of Physicians; 2010.

[11] Tobacco Free Kids. Tobacco Control Policy Fact Sheet. Bangladesh: Smoke Free Places. *Tobacco Control Laws* 2015 http://www.tobaccocontrollaws.org/. Accessed May 28, 2017.

[12] Mbulo L, Palipudi KM, Andes L, et al Secondhand smoke exposure at home among one billion children in 21 countries: Findings from the Global Adult Tobacco Survey (GATS). Tob Control. 2016;25(e2):e95–e100. doi:10.1136/tobaccocontrol-2015-052693. 26869598 10.1136/tobaccocontrol-2015-052693 PMC5488799

[13] Baxi R, Sharma M, Roseby R, et al Family and carer smoking control programmes for reducing children’s exposure to environmental tobacco smoke. Cochrane Database Syst Rev. 2014;3:CD001746 Accessed August 22, 2014. doi:10.1002/14651858.CD001746.pub3. 24671922 10.1002/14651858.CD001746.pub3

[14] Daly JB, Mackenzie LJ, Freund M, Wolfenden L, Roseby R, Wiggers JH Interventions by health care professionals who provide routine child health care to reduce tobacco smoke exposure in children: A review and meta-analysis. JAMA Pediatr. 2016;170(2):138–147. doi:10.1001/jamapediatrics.2015.3342 26719991 10.1001/jamapediatrics.2015.3342

[15] Rosen LJ, Myers V, Winickoff JP, Kott J Effectiveness of interventions to reduce tobacco smoke pollution in homes: A systematic review and meta-analysis. Int J Environ Res Public Health. 2015;12(12):16043–16059. doi:10.3390/ijerph121215038. 26694440 10.3390/ijerph121215038 PMC4690974

[16] Huque R, Dogar O, Cameron I, Thomson H, Amos A, Siddiqi K Children learning about second-hand smoking: A feasibility cluster randomized controlled trial. Nicotine Tob Res. 2015;17(12):1465–1472. doi:10.1093/ntr/ntv015. 25634936 10.1093/ntr/ntv015

[17] Siddiqi K, Huque R, Jackson C, et al Children learning about secondhand smoke (CLASS II): Protocol of a pilot cluster randomised controlled trial. BMJ Open. 2015;5(8):e008749. doi:10.1136/bmjopen-2015-008749. 10.1136/bmjopen-2015-008749 PMC4550726 26307620

[18] Bennett AE, Power TJ, Eiraldi RB, Leff SS, Blum NJ Identifying learning problems in children evaluated for ADHD: The academic performance questionnaire. Pediatrics. 2009;124(4):e633–e639. doi:10.1542/peds.2009-0143. 19736265 10.1542/peds.2009-0143 PMC2864516

[19] Michie S, Richardson M, Johnston M, Abraham C, Francis J, Hardeman W The behavior change technique taxonomy (v1) of 93 hierarchically clustered techniques: Building an international consensus for the reporting of behavior change interventions. Ann Behav Med. 2013;46(1):81–95. doi:10.1007/s12160-013-9486-6. 10.1007/s12160-013-9486-6 23512568

[20] Chauhan AJ, Inskip HM, Linaker CH, et al Personal exposure to nitrogen dioxide (NO2) and the severity of virus-induced asthma in children. Lancet. 2003;361(9373):1939–1944. doi:10.1016/S0140-6736(03)13582-9. 12801737 10.1016/S0140-6736(03)13582-9 PMC7112409

[21] Consortium BC. Spirometry in Practice: A Practical Guide to Using Spirometry in Primary Care. London: British Thoracic Society; 2005.

[22] Boston RC, Sumner AE STATA: A statistical analysis system for examining biomedical data. Adv Exp Med Biol. 2003;537:353–369. 14995047 10.1007/978-1-4419-9019-8_23

[23] Ainsworth H, Shah S, Ahmed F, et al Muslim communities learning about second-hand smoke (MCLASS): Study protocol for a pilot cluster randomised controlled trial. Trials. 2013;14(1):295. doi:10.1186/1745-6215-14-295. 24034853 10.1186/1745-6215-14-295 PMC3847687

[24] Burström K, Bartonek Å, Broström EW, Sun S, Egmar AC EQ-5D-Y as a health-related quality of life measure in children and adolescents with functional disability in Sweden: Testing feasibility and validity. Acta Paediatr. 2014;103(4):426–435. doi:10.1111/apa.12557. 24761459 10.1111/apa.12557

[25] Huang CY, Reisch LA, Gwozdz W, et al Pester power and its consequences: Do European children’s food purchasing requests relate to diet and weight outcomes?Public Health Nutr. 2016;19(13):2393–2403. doi:10.1017/S136898001600135X. 27297518 10.1017/S136898001600135X PMC10270966

[26] Rosen LJ, Myers V, Hovell M, Zucker D, Ben Noach M Meta-analysis of parental protection of children from tobacco smoke exposure. Pediatrics. 2014;133(4):698–714. doi:10.1542/peds.2013-0958. 24664094 10.1542/peds.2013-0958

[27] Baxter S, Blank L, Everson-Hock ES, et al The effectiveness of interventions to establish smoke-free homes in pregnancy and in the neonatal period: A systematic review. Health Educ Res. 2011;26(2):265–282. doi:10.1093/her/cyq092. 21273185 10.1093/her/cyq092

[28] Priest N, Roseby R, Waters E, et al Family and carer smoking control programmes for reducing children’s exposure to environmental tobacco smoke. Cochrane Database Syst Rev. 2008;4:CD001746. doi:10.1002/14651858.CD001746.pub2. 18843622 10.1002/14651858.CD001746.pub2

[29] Elder JP, Perry CL, Stone EJ, et al Tobacco use measurement, prediction, and intervention in elementary schools in four states: The CATCH Study. Prev Med. 1996;25(4):486–494. doi:10.1006/pmed.1996.0080. 8812826 10.1006/pmed.1996.0080

