# Supplementary material for: Children Learning About Secondhand Smoke (CLASS II): A Pilot Cluster Randomized Controlled Trial
Source: Nicotine Tob Res. 2018 May 16;21(5):670–7. doi: 10.1093/ntr/nty090 (PMC6468126; doi:10.1093/ntr/nty090)
Supplement: Supplementary Material [file nty090_suppl_supplementary_material.docx]

**Supplementary table 1: Descriptive statistics of the educational outcomes at the cluster level at baseline, 2-month, 6-month, and 12-month follow-ups**

|  |  | **Intervention** | | | | **Control** | | | |
| --- | --- | --- | --- | --- | --- | --- | --- | --- | --- |
|  |  | **Baseline** | **2 months** | **6 months** | **12 months** | **Baseline** | **2 months** | **6 months** | **12 months** |
| Reading | Well above average | 0.27  (0.24) | 0.33  (0.224) | 0.41  (0.355) | 0.43  (0.309) | 0.31  (0.168) | 0.41  (0.258) | 0.42  (0.25) | 0.47  (0.354) |
|  | At or somewhat above average | 0.40  (0.16) | 0.53  (0.264) | 0.49  (0.318) | 0.40  (0.245) | 0.29  (0.177) | 0.39  (0.212) | 0.49  (0.246) | 0.45  (0.303) |
|  | Somewhat below average | 0.32  (0.219) | 0.14  (0.15) | 0.09  (0.125) | 0.16  (0.258) | 0.37  (0.182) | 0.17  (0.183) | 0.09  (0.094) | 0.07  (0.087) |
|  | Well below average | 0.00  (0.011) | _ | _ | _ | 0.03  (0.055) | 0.03  (0.063) | _ | 0.00  (0.018) |
| Comprehension | Well above average | 0.16  (0.142) | 0.27  (0.228) | 0.28  (0.36) | 0.24  (0.166) | 0.17  (0.159) | 0.27  (0.201) | 0.43  (0.282) | 0.13  (0.111) |
| Perform Maths Calculations | At or somewhat above average | 0.41  (0.2) | 0.43  (0.19) | 0.47  (0.317) | 0.45  (0.187) | 0.3  (0.063) | 0.46  (0.109) | 0.39  (0.151) | 0.52  (0.268) |
|  | Somewhat below average | 0.42  (0.287) | 0.3  (0.181) | 0.24  (0.222) | 0.30  (0.266) | 0.46  (0.145) | 0.25  (0.168) | 0.18  (0.175) | 0.31  (0.223) |
|  | Well below average | 0.013  (0.021) | 0.01  (0.013) | 0.00  (0.012) | - | 0.08  (0.143) | 0.03  (0.063) | - | 0.04  (0.106) |
|  | Well above average | 0.19  (0.183) | 0.23  (0.171) | 0.23  (0.239) | 0.31  (0.228) | 0.16  (0.155) | 0.26  (0.14) | 0.27  (0.205) | 0.34  (0.244) |
|  | At or somewhat above average | 0.37  (0.131) | 0.43  (0.169) | 0.36  (0.095) | 0.43  (0.127) | 0.27  (0.049) | 0.43  (0.106) | 0.52  (0.185) | 0.45  (0.18) |
|  | Somewhat below average | 0.43  (0.233) | 0.33  (0.218) | 0.39  (0.281) | 0.25  (0.203) | 0.54  (0.141) | 0.28  (0.152) | 0.19  (0.13) | 0.19  (0.092) |
|  | Well below average | 0.00  (0.011) | - | 0.02  (0.029) | 0.02  (0.049) | 0.03  (0.054) | 0.03  (0.063) | 0.00  (0.014) | 0.02  (0.036) |
| Word problems | Well above average | 0.20  (0.223) | 0.26  (0.238) | 0.16  (0.127) | 0.22  (0.15) | 0.15  (0.16) | 0.22  (0.159) | 0.32  (0.193) | 0.26  (0.222) |
|  | At or somewhat above average | 0.32  (0.114) | 0.43  (0.215) | 0.54  (0.141) | 0.5  (0.143) | 0.26  (0.101) | 0.48  (0.167) | 0.41  (0.1) | 0.46  (0.207) |
|  | Somewhat below average | 0.45  (0.243) | 0.31  (0.205) | 0.24  (0.169) | 0.24  (0.147) | 0.55  (0.157) | 0.28  (0.178) | 0.27  (0.195) | 0.27  (0.179) |
|  | Well below average | 0.03  (0.029) | 0.00  (0.012) | 0.06  (0.142) | 0.04  (0.099) | 0.04  (0.066) | 0.03  (0.063) | - | 0.01  (0.020) |
| Writing (Short Stories) | Well above average | 0.21  (0.14) | 0.27  (0.22) | 0.21  (0.194) | 0.34  (0.208) | 0.14  (0.16) | 0.17  (0.152) | 0.33  (0.173) | 0.30  (0.264) |
|  | At or somewhat above average | 0.44  (0.186) | 0.49  (0.171) | 0.39  (0.155) | 0.45  (0.15) | 0.24  (0.12) | 0.55  (0.223) | 0.44  (0.135) | 0.46  (0.236) |
|  | Somewhat below average | 0.33  (0.183) | 0.22  (0.106) | 0.38  (0.247) | 0.19  (0.156) | 0.54  (0.171) | 0.24  (0.168) | 0.21  (0.115) | 0.20  (0.131) |
|  | Well below average | 0.02  (0.020) | 0.01  (0.015) | 0.02  (0.048) | 0.02  (0.049) | 0.08  (0.138) | 0.03  (0.062) | 0.03  (0.041) | 0.03  (0.069) |
| * Mean (SD) are reported at the cluster level. | | | | | | | | | |

**Supplementary table 2: Descriptive statistics of the respiratory symptom diary scores at the cluster level**

|  | **Intervention** | | | **Control** | | |
| --- | --- | --- | --- | --- | --- | --- |
| **Outcomes** | **0-2 months** | **3-6 months** | **7-12 months** | **0-2 months** | **3-6 months** | **7-12 months** |
| Number of Clusters | 6 | 6 | 6 | 6 | 6 | 6 |
| Number of Children Available | 209 | 217 | 215 | 194 | 203 | 201 |
| Number of Children who Completed Diary | 147 | 129 | 120 | 139 | 76 | 129 |
| Number of Children with at least one score above threshold | 135 | 103 | 103 | 119 | 69 | 116 |
| % of children with at least one score above threshold (SD) | 0.92  (0.048) | 0.84  (0.175) | 0.84  (0.152) | 0.82  (0.269) | 0.92  (0.15) | 0.88  (0.061) |
| Average number of weeks above threshold (SD) | 5.07  (1.3) | 8.47  (2.27) | 10.2  (3.48) | 5.05  (1.85) | 10  (3.57) | 10.2  (4.15) |

**Supplementary table 3a: Numbers and percentages reporting no problems on EQ-5D-Y by control [C] and intervention [I]**

|  | **Baseline**  **(n=481)** | | **2 months**  **(n=433)** | | **6 months**  **(n=449)** | | **12 months**  **(n=446)** | |
| --- | --- | --- | --- | --- | --- | --- | --- | --- |
|  | C  (n=236) | I  (n=245) | C  (n=209) | I  (n=224) | C  (n=217) | I  (n=232) | C (n=216) | I  (n=230) |
| Reporting no problems (n) | 112 | 145 | 128 | 126 | 113 | 122 | 118 | 130 |
| % reporting no problems | 47.4% | 59.2% | 61.2% | 56.2% | 52.1% | 52.6% | 54.6% | 56.5% |

**Supplementary table 3b: Mean (SD) health care utilisation by treatment group**

|  | **Baseline**  (Previous 6 months) | | **2 months**  (Previous 2 months) | | **6 months**  **(**Previous 4 months) | | **12 months**  (Previous 6 months) | |
| --- | --- | --- | --- | --- | --- | --- | --- | --- |
|  | **Control** | **Intervention** | **Control** | **Intervention** | **Control** | **Intervention** | **Control** | **Intervention** |
| Appointments with doctor at health centre/clinic/hospital | .49  (.236) | .57  (1.184) | .11  (.543) | .31  (1.220) | .26  (.552) | .33  (.861) | .38  (.725) | .17  (.472) |
| Appointments with doctor at home | .05  (.323) | .02  (.127) | .04  (.431) | .01  (.115) | .02  (.178) | .02  (.193) | .01  (.152) | .02  (.131) |
| Appointments with nurse at health centre/clinic/hospital | .10  (.406) | .11  (.462) | .03  (.238) | .04  (.339) | .02  (.178) | .03  (.209) | .07  (.391) | .02  (.161) |
| Appointments with nurse at home | .06  (.619) | .02  (.201) | .00  (.069) | .00  (.067) | .00  (.000) | .00  (.000) | .01  (.096) | .00  (.000) |
| Outpatient visits | .14  (.714) | .14  (.476) | .02  (.169) | .10  (.797) | .03  (.201) | .05  (.275) | .11  (.401) | .10  (.483) |
| Hospital admission | .03  (.193) | .06  (.295) | .00  (.000) | .01  (.096) | .00  (.068) | .01  (.081) | .01  (.117) | .03  (.096) |
| Emergency visits | .03  (.223) | .04  (.283) | .00  (.000) | .02  (.209) | .00  (.068) | .01  (.081) | .01  (.096) | .03  (1.85) |
| Prescriptions | .44  (.895) | .56  (.946) | .12  (.480) | .22  (.822) | .28  (.558) | .29  (.655) | .61  (1.271) | .37  (.966) |

**Supplementary table 3c: Percentage of zero health care utilisation by treatment group**

|  | **Baseline** | | **2 months** | | **6 months** | | **12 months** | |
| --- | --- | --- | --- | --- | --- | --- | --- | --- |
|  | **Control**  **n=236** | **Intervention**  **n=245** | **Control**  **n=209** | **Intervention**  **n=224** | **Control**  **n=218** | **Intervention**  **n=232** | **Control**  **n=216** | **Intervention**  **n=230** |
| Appointments with doctor at health centre/clinic/hospital | 72.03 | 70.61 | 92.82 | 85.27 | 78.44 | 81.47 | 72.69 | 85.65 |
| Appointments with doctor at home | 97.03 | 98.37 | 98.09 | 98.66 | 98.17 | 98.28 | 99.07 | 98.26 |
| Appointments with nurse at health centre/clinic/hospital | 93.22 | 93.06 | 98.09 | 97.77 | 98.17 | 97.84 | 95.37 | 98.70 |
| Appointments with nurse at home | 97.88 | 98.37 | 99.52 | 99.55 | 100 | 100 | 99.07 | 100 |
| Outpatient visits | 94.07 | 90.20 | 98.56 | 91.96 | 97.25 | 96.12 | 91.67 | 94.35 |
| Hospital admission | 97.46 | 95.51 | 100 | 98.21 | 99.54 | 99.14 | 98.61 | 96.96 |
| Emergency visits | 97.46 | 97.55 | 100 | 98.21 | 99.54 | 99.14 | 99.07 | 97.83 |
| Prescriptions | 72.88 | 64.90 | 91.87 | 84.38 | 77.06 | 80.17 | 71.30 | 80.00 |
